# Supplementary material for: Mapping the evolution of fertility support policies in China: A content and instrumental analysis
Source: PLoS One. 2025 Oct 9;20(10):e0332137. doi: 10.1371/journal.pone.0332137 (PMC12510515; doi:10.1371/journal.pone.0332137)
Supplement: S1 Appendix — (ZIP) [file pone.0332137.s001.zip › S1 Appendix. 226 original policy documents/201-共青团中央、民政部、国家卫生计生委关于印发《关于进一步做好青年婚恋工作的指导意见》的通知(FBM-CLI-4-301999).doc]

共青团中央、民政部、国家卫生计生委关于印发《关于进一步做好青年婚恋工作的指导意见》的通知

**发布部门：** [共青团中央](https://www.pkulaw.com/law/chl?Aggs.IssueDepartment=70402) [民政部](https://www.pkulaw.com/law/chl?Aggs.IssueDepartment=60208) [国家卫生和计划生育委员会(已撤销)](https://www.pkulaw.com/law/chl?Aggs.IssueDepartment=60319) [机构沿革](javascript:void(0))

**发布日期：**2017.09.04

**实施日期：**2017.09.04

**时效性：** [现行有效](https://www.pkulaw.com/law/chl?Aggs.TimelinessDic=01)

**效力级别：** [部门规范性文件](https://www.pkulaw.com/law/chl?Aggs.EffectivenessDic=XE0302)

**法规类别：** [婚姻登记](https://www.pkulaw.com/law/chl?Aggs.Category=02203)

共青团中央、民政部、国家卫生计生委关于印发《关于进一步做好青年婚恋工作的指导意见》的通知

各省、自治区、直辖市团委、民政厅（局）、卫生计生委，中央军委政治工作部组织局群团处，全国铁道团委，全国民航团委，中直机关团工委，中央国家机关团工委，中央金融团工委，中央企业团工委，新疆生产建设兵团团委、民政局、卫生局、人口计生委：
　　为贯彻落实《[中长期青年发展规划（2016-2025年）](https://www.pkulaw.com/chl/c9ba542878e8ad32bdfb.html)》，推动服务青年婚恋工作，特制定《关于进一步做好青年婚恋工作的指导意见》。现印发给你们，请结合实际认真贯彻执行。

共青团中央
民政部
国家卫生计生委
2017年9月4日

　　关于进一步做好青年婚恋工作的指导意见

　　恋爱成家是青年的人生大事和普遍需求。做好青年婚恋工作，不仅直接影响青年的健康发展，也关系到社会的和谐稳定。今年4月，中共中央、国务院印发《[中长期青年发展规划（2016-2025年）](https://www.pkulaw.com/chl/c9ba542878e8ad32bdfb.html)》（以下简称[《规划》](https://www.pkulaw.com/chl/c9ba542878e8ad32bdfb.html)），将青年婚恋作为青年发展的重要领域，明确了目标任务，制定了发展措施。为推动[《规划》](https://www.pkulaw.com/chl/c9ba542878e8ad32bdfb.html)有效实施，健全青年婚恋的社会支持体系，现制定以下指导意见。
　　一、思路和目标
　　贯彻落实[《规划》](https://www.pkulaw.com/chl/c9ba542878e8ad32bdfb.html)关于青年婚恋的工作部署，健全政府部门、群团组织、社会力量齐抓共促青年婚恋工作的协同推进机制，加强青年婚恋观、家庭观的教育和引导，培育诚信度较高、适合青年特点需求的多样化青年婚恋服务项目，开展青年性健康教育和优生优育宣传教育，不断优化完善青年婚恋生育相关政策，推动青年婚恋观念更加文明、健康、理性，青年婚姻家庭和生殖健康服务水平进一步提升，青年相关法定权利得到更好保障。

　　二、基本原则
　　1.突出价值引领。把服务青年需求与引领青年践行社会主义核心价值观结合起来，在帮助青年解决交友、择偶、婚姻实际问题的同时，强化青年对情感生活的尊重意识、诚信意识和责任意识，加强对青年婚恋观、家庭观的教育和引导。
　　2.强化公益导向。鼓励面向青年开展的公益性婚恋交友服务，打造诚信度高、公益性强的青年交友信息平台，提高青年获取婚恋婚育权益维护和公共服务的可及性，推动形成关心青年婚恋、促进青年成长的良好社会氛围。
　　3.坚持青年为本。适应青年多样化需求和差异化特点，拓宽青年交友交流的渠道，培育富有实效、内涵、特色的服务项目，扩大青年反映呼声和参与评价的渠道，健全以青年满意度为核心的婚恋服务评价体系。
　　4.务求工作实效。以市、县（区）为工作主体，就近就便开展青年婚恋工作。密切政府部门、群团组织、社会力量的协同合作，促进基层婚恋婚育服务水平的提升，共同推动相关政策法规的完善和执行。

　　三、工作内容
　　1.弘扬文明婚恋风尚。将倡树婚恋文明新风融入培育和践行社会主义核心价值观工作体系，大力倡导文明、健康、理性的婚恋观念，形成积极健康的婚恋舆论导向。争取宣传、文化部门和新闻媒体的支持，在开展精神文明创建活动中，注重选树彰显正面婚恋观念的典型，推动协调新闻媒体、文艺社团、文化公司等打造积极向上的婚恋影视作品、新闻栏目和文化产品。各级团组织要配合相关政府部门，积极开展融入婚恋文明内涵的社区服务、法律援助、文化宣传等群众性活动，倡导家庭美德和文明节俭的婚庆文化，宣传婚姻政策及相关法规，丰富婚恋文明的时代内涵和实践载体。
　　2.加强婚恋咨询与指导。加强对青年恋爱交友过程、婚姻家庭生活的心理和行为指导，在帮助青年解决实际问题的同时，加强青年价值引导、促进青年健康发展。依托民政、卫生计生、司法等专业力量，加强对专兼职心理咨询师和婚恋咨询、婚姻调解等社工队伍的培训，引导社会力量开展专业化咨询，丰富社区服务机构、医疗机构、婚介机构的咨询功能，提高青年婚恋咨询和指导的可及性和专业性。各级团组织要结合青年特点开展服务和引导工作，通过线上咨询、婚恋讲堂、面对面沟通等多种方式，帮助青年解除思想压力和心理困惑，提高青年社会融入、情绪管理、情感经营能力，引导青年端正择偶观念和家庭观念。
　　3.普及性健康和优生优育知识。开展青年性健康、婚前保健和生育知识的教育宣传活动，促进青年生殖健康水平进一步提升。推动有条件的学校推广性健康和婚前保健课程，加强性教育和婚前保健服务师资队伍建设。以世界人口日、五四青年节等为契机，大力开展性健康、婚前保健和婚育文明主题宣传活动，利用公共场所、结婚登记场所、网络媒体，走进企业、社区、学校等，开展形式多样的宣讲、服务、咨询活动，重点加强进城务工青年群体的公益服务和知识普及，增强青年主动预防和减少不当性行为的意识和能力。加强基层青年婚恋服务阵地建设，依托各类“青年之家”、城乡社区服务机构等开展婚育文化教育，引导青年主动实施婚前、孕前和产前医学检查。
　　4.培育公益性婚恋服务项目。更好适应青年交友、相亲、婚介、婚庆的多元多样需求，动员和支持基层团组织培育有形有效的服务项目。组织单身青年参加文体娱乐、兴趣培养、技能提升、社会服务等健康向上的集体活动，拓展青年社会交往的广度和深度，丰富青年交友择偶的机会。缓解青年婚恋经济压力，通过举办集体婚礼、优化婚庆服务、提供恋爱交友便利条件等，为青年提供贴心暖心的服务。加大对重点青年群体的服务力度，针对大龄未婚青年、进城务工青年、农村青年、青年官兵、部分行业青年等的婚恋难题和不同特点，尊重差异与个性，注重分类精准施策，提高服务针对性和有效性。
　　5.拓展线上线下服务平台。整合民政部门、团组织、市场机构、社会组织的阵地和平台资源，打造一批便于青年参与、服务实效显著、有较高诚信度的婚恋公益服务平台。拓展实体服务阵地，为青年开展身份认证、婚恋咨询、联谊交流、交友约会等活动提供便利化精准化的服务。拓展线上服务平台，依托“青年之声”等团属媒体、团组织微博、微信公众号等，拓展婚恋咨询、婚恋调查、活动发布、活动报名的线上渠道，探索开发具备信息搜索、匹配推介、地图服务等功能的移动互联婚恋交友平台，将线上优势与线下服务深度融合，扩大服务覆盖、优化青年体验。
　　6.促进婚恋市场规范发展。建立健全婚恋交友信息平台、婚介婚庆服务机构的行业标准体系和监测评估体系。协调推动工商、工信、公安、网监、机关职能等部门的协同联动，推动实名认证和实名注册在婚恋交友平台的严格执行，加强对个人用户信息保护的监督执法，依法整顿婚介服务市场，严厉打击婚托、婚骗等违法婚介行为。加强婚恋市场秩序的日常监管、婚恋服务质量的动态评估，拓展群团组织、青年、第三方机构参与评价的渠道。
　　7.保障青年婚姻和生育合法权益。配合公安机关，依法处理包办、买卖等侵犯青年婚姻自由的行为。维护女性青年生育权益，落实女性青年在怀孕、生育和哺乳期间依法享有的物质和假期等方面的各项权利。对育龄劳动歧视、家庭暴力、财产纠纷等侵犯青年婚姻和生育合法权益的，积极配合相关部门，动员律师等专业力量，为青年提供必要帮助。

　　四、组织保障
　　1.加强组织领导。民政部、卫生计生委、共青团中央是落实[《规划》](https://www.pkulaw.com/chl/c9ba542878e8ad32bdfb.html)青年婚恋工作的主要部门，积极构建协同联动的工作机制，共同推动青年婚恋工作深入开展。省级民政部门、卫生计生部门、团委，主要负责本地区服务青年婚恋交友工作的组织领导，要重点做好思路谋划、统筹指导、支持保障等工作。市、县（区）民政部门、卫生计生部门、团委是青年婚恋工作的实施主体，要结合实际，突出重点，面向青年做好具体服务和引导工作，要做出特色、务求实效。
　　2.形成社会合力。要推动青年婚恋交友服务体系的社会化发展，构建党政部门、群团组织、社会力量合力推动青年婚恋工作的良好格局。要发挥共青团协调作用，组建政府部门、行业协会、高校、社科机构等领域有关人士组成的专家团队，为青年婚恋工作发挥智库作用。要深化区域化团建工作合作，推动各级各类团组织服务青年婚恋交友的工作整合。要拓展基层团组织服务青年婚恋工作的社会化合作平台，支持婚恋领域青年组织开展联谊、婚介、交友等特色服务项目。
　　3.强化政策保障。要加强青年婚恋状况的调查研究和关键数据的监测评估，为开展青年婚恋工作提供科学有力的依据。发挥民政部门、卫生计生部门的职能作用和专业优势，加强顶层设计，出台政策措施，为青年获取婚育服务提供便利。发挥共青团桥梁纽带作用，依托“共青团与人大代表、政协委员面对面”等渠道，及时反映青年呼声，促进青年婚恋相关政策的优化完善。

© 北大法宝：（[www.pkulaw.com](https://www.pkulaw.com/)) 专业提供法律信息、法学知识和法律软件领域各类解决方案。北大法宝为您提供丰富的参考资料，正式引用法规条文时请与标准文本核对。
欢迎查看所有[产品和服务](https://www.pkulaw.com/helps/2.html)。法宝快讯：[如何快速找到您需要的检索结果？](https://www.pkulaw.com/helps/70.html)[法宝](https://www.pkulaw.com/helps/69.html)V6有何新特色？

扫描二维码阅读原文

**原文链接：**<https://www.pkulaw.com/chl/3751db6e28067701bdfb.html>
